# Aging results in iron accumulations in the non-human primate choroid of the eye without an associated increase in zinc, copper or sulphur

**DOI:** 10.1007/s10534-018-0147-x

**Published:** 2018-10-10

**Authors:** M. Ugarte, K. Geraki, G. Jeffery

**Affiliations:** 1 Moorfields Eye Hospital NHS Foundation Trust, London, EC1V 2PD UK; 2 NIHR Biomedical Research Centre, Moorfields Eye Hospital and UCL Institute of Ophthalmology, London, UK; 3 I18, Diamond Light Source, Harwell Science and Innovation Campus, Fermi Ave, Didcot, Oxfordshire OX11 0DE UK; 4 UCL Institute of Ophthalmology, 11-43 Bath St, London, EC1V 9EL UK

**Keywords:** Age-related macular degeneration, Retina, Trace elements, Choroid, Iron, Zinc, Copper

## Abstract

We present further analyses of a previous experiment published in 2016 where the distribution, concentration and correlation of iron, zinc, copper and sulphur in the choroid of the eye in young and aged old world primates (*Macaca fascicularis*) was studied with synchrotron X-ray fluorescence with a 2 μm resolution. The results indicate that iron accumulates in hotspots in the choroid with age with fluorescence intensity ranging from 2- to 7-fold (1002–3752 ppm) the mean level in the choroidal stroma (500 ppm) and maximum iron levels in blood vessel lumina. Iron hotspots with iron ppm > 1000 preferentially contained Fe^3+^ as demonstrated by Perls staining. There was a strong spatial co-localisation and correlation between copper and zinc (Pearson’s correlation coefficient 0.97), and both elements with sulphur in the choroid of young animals. However, these are reduced in the choroid of aged animals and lost in the iron hotspots. The lack of proportional co-distribution suggests that iron accumulation does not induce a concomitant increase in zinc, copper or zinc-, copper-metalloproteins. It is possible that the iron hotspots are ferritin or hemosiderin molecules loaded with Fe^3+^ in stable, insoluble, non-toxic complexes without a significant oxidative environment.

## Introduction

Iron is a trace element essential for normal cellular and tissue function, being part of heme and non-heme proteins. It is a key component of haemoglobin, myoglobin, cytochromes and iron-containing metalloenzymes involved in vital metabolic and biosynthetic biological pathways (Jellinger 2013). The labile reactive form of iron enhances oxidative stress and can result in free radical induced cell death (Sfera et al. 2017). Its homeostasis is, therefore, tightly regulated by iron transporters and sequestration in bioavailable non-toxic form bound to melanin (Andrzejczyk and Buszman 1992), or in ferritin and hemosiderin, the major cellular iron storage proteins (Winter et al. 2014).

The content and distribution of iron in the retina and choroid have been analysed in relation to ageing and age-related macular degeneration (AMD), the most common blinding condition in the population over the age of 50 (Stevens et al. 2013). We recently demonstrated that iron-rich deposits accumulate in the non-human primate choroid with age (Ugarte et al. 2016). Other groups have described accumulation of iron in Bruch’s membrane and retinal pigment epithelial cells in donor eyes with AMD using histochemical methods and analytical electron microscopy (Biesemeier et al. 2015; Hahn et al. 2003; Wong et al. 2007).

It has long been known that the homeostasis of iron, zinc and copper are closely interrelated (Ugarte et al. 2013). They can compete for the same ligand and displace each other directly. Iron accumulation in the brain (Yoshida et al. 2017) and liver (Ono et al. 2015) in systemic conditions with iron overload results in parallel copper and sulphur deposition. The active redox state in iron-rich deposits is thought to result in an increased requirement for copper and cuproproteins (e.g. multicopper oxidases, Cu/Zn superoxide dismutase) to regulate the iron-induced oxidative stress. However, no studies have been conducted to evaluate whether iron increases in the retina and choroid are concomitant with changes in the content of sulphur, copper and/or zinc.

This study includes further analysis of the data acquired in a previous experiment published by our group in 2016 (Ugarte et al. 2016). In order to visualise total iron, zinc, copper and sulphur simultaneously, we used synchrotron X-ray fluorescence (SXRF). Its high sensitivity, spatial resolution and ability to quantify elemental levels make it more powerful than iron histochemical stains or conventional quantitative methods, such as atomic absorption spectrometry or inductively coupled plasma mass spectrometry (ICPMS). Subtle focal changes in trace elements content and/or distribution would be extremely difficult, if not impossible, to detect by other means. Staining methods only identify chemically reactive iron and fail to provide information about concentration. Conventional quantitative methods, on the other hand, lack spatial resolution. We assessed co-localisation of different trace elements visually by false colour overlays of two specific elements and quantitatively by evaluating the correlation of concentrations corrected for elemental sensitivity on a pixel-by-pixel basis and calculating the Pearson’s correlation coefficient. This allowed us to address the question of whether iron-rich deposits share the same localisation with sulphur, copper or zinc levels different from the average choroidal stroma. It is important to understand the normal distribution of iron, copper and zinc, their relationship and the reaction of choroidal tissue to iron accumulation in aging to understand potentially important changes in a tissue that is key to outer retinal function. Such a study will also provide a platform for an analysis of such deposits in disease, potentially including AMD.

## Methods

Tissue preparation and synchrotron analysis was as previously described (Ugarte et al. 2016).

### Tissue preparation

All animal procedures were undertaken under local ethical procedures, in line with the UK Home Office regulations [Animals (Scientific Procedures) Act 1986] and the tenets of the ARVO statement on the Use of Animals in Ophthalmic and Vision Research (http://arvoprod.serverside.net) (ARVO 2018). Trace element contamination was minimised by using analytical grade reagents and handling samples in clean environments with titanium tools. Tissue from old world primate model (*Macaca fascicularis*) was used. All animals were provided with the same commercially available primate diet and water ad libitum, and were exposed exactly to the same environment. Age was the only variable that differed between the groups. All macaques were clinically healthy during the study period. They did not receive any trace element supplements or blood transfusions. Unnatural constraint (laboratory rearing versus free ranging) is unlikely to have affected trace element metabolism. Primary animal use was for purpose other (i.e. vaccine testing) than the experiments described here. Four young (4–5 years) and 5 aged (15–16 years) male animals were sedated early in the morning with 0.1 mg/kg Dormitol and 200 mg/kg Ketamine intramuscularly, bled out and humanely killed with 2.5 ml Nembutal (50 mg sodium pentobarbital per ml) intracardially. After removal of the eyeballs, the cornea, iris and lens were dissected and the remaining tissues were fixed in 4% paraformaldehyde for less than 48 h. After cryopreservation in 25% sucrose tissue samples were embedded in optimum cutting temperature Tissue-Teks compound. Thirty μm-thick sections of retina and choroid from the macular region were cut with a cryostat, placed on thin film and allowed to air dry.

### Synchrotron X-ray fluorescence

Within a few days of cutting the sections, the tissue samples were imaged at I18 the Microfocus Spectroscopy beamline (Mosselmans et al. 2009) at the synchrotron Diamond Light Source (Harwell Science and Innovation Campus, UK) (http://www.diamond.ac.uk) (Diamond 2018). The distribution of total iron, zinc and copper was mapped at micrometer level resolution in air at room temperature.

Kirkpatrick Baez mirrors were used for focusing the X-ray beam. The energy used to excite the elements of interest was 11 keV. A silicon drift detector (SGX) was used for the collection of the fluorescence signal. A beam of 2 × 2 μm^2^ (H × V) was employed to map areas of approximate 200 × 100 μm^2^ on each sample focusing in the macular area (central region of the eye posterior pole). Processing of the raw data involved peak fitting and background removal. Quantitative calculation of the trace element concentrations was carried out by measuring a reference material (AXO, Dresden GmbH) in the same conditions as the samples. This reference material is one of the most frequently used for micro-XRF because of its high uniformity, stability and detailed characterization by the manufacturers. It is composed of nm-thick layers of metals with known numbers of atoms per unit area. We used PyMca software (Solé et al. 2007) to model both our samples and the reference material in terms of main composition, density and thickness with corrections of the fluorescence signal for the two different matrices. The characteristic photons from each element detected at each pixel were converted to concentration values with units of ppm (µg/g dry weight) therefore the contrast of the resulting maps represents concentration gradients. The maps were imported as tiff images in ImageJ (ImageJ 2018) (https://imagej.nih.gov/ij/).

We carried out two different types of comparisons: (a) effect size by the standardized difference between two means (young and aged) and (b) comparisons of measures of association between variables (i.e. Pearson’s correlation coefficient, the degree of linear relationship between two quantitative variables-trace elements concentrations). We determined that a number of 4–5 animals in each group was sufficient to ensure that the study had acceptable power to detect an effect size of 0.5 (considered “medium”) and a p < 0.05.

### Co-localisation

In order to investigate whether specific trace elements overlapped spatially or were distributed proportionally, we carried out qualitative and quantitative co-localisation analysis. Visual co-localisation was assessed subjectively by comparing the relative distribution of each trace element side by side in individual maps, as well as in merged maps obtained using the multiple-channel fluorescence images display on PyMca. The degree of co-localisation was quantified by plotting the fluorescence intensity of one metal against a second metal for each pixel. Clustering of the points around a straight line would be consistent with proportional co-distribution. Quantitative evaluation was performed by using the Pearson’s correlation coefficient, whose value depends on a simple linear relationship with high figures showing strong co-localisation. Careful consideration of factors known to affect the Pearson’s correlation coefficient (i.e. noise, background, intensity variability) was taken. High signal-to-noise ratios were always confirmed before we carried out the analyses and for this the section thickness had to be 30 μm.

### Staining

Chemically reactive iron was identified by using the chromophore, potassium ferrocyanide (Perls reagent), which after reacting with ferric ions (Fe^3+^) produces Prussian blue (PB). In contrast to the SXRF, which maps total iron. Perls staining shows non-heme–iron such as ferritin and hemosiderin-bound Fe^3+^.

The presence of microglia and macrophage was assessed by staining for Iba-1 marker. Ten µm thick sections were kept for 1 h in 5% Normal Donkey serum in 0.3% Triton X-100 in PBS, pH 7.4, followed by an overnight incubation with rabbit polyclonal primary antibody to Iba-1 (1:1000, A. Menarini Diagnostics, UK) in 1% Normal Donkey Serum in 0.3% (v/v) Triton X-100 in PBS. The secondary antibody conjugated with Alexa fluor 488 (Invitrogen Molecular Probes, UK) made up in 2% Normal Donkey Serum in 0.3% Triton X-100 in PBS (1:2000) was added to the sections and incubated for 1 h at room temperature. We omitted the primary antibody in negative controls. DAPI (4′,6-diamidino-2-phenylindole) staining was used for nuclear acid (nuclear) labelling.

## Results

### Choroid layers, structures, function

**Fig. 1:**
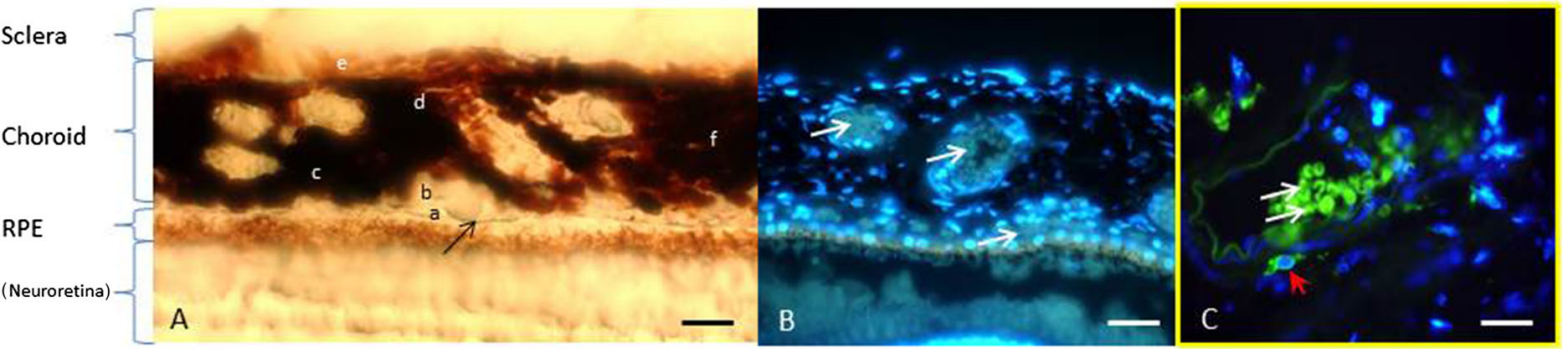
Images of old world primate (*Macaca fascicularis*) choroid, the highly pigmented vascular structure of the eye, from the macular area. **a** Bright field image showing the choroid between the retina and the sclera with its 5 distinct regions: (*a*) Bruch’s membrane (a basal membrane) (black arrow), (*b*) choriocapillaris (network of wide calibre capillaries), (*c*) Sattler’s layer (small, medium sized vessels), (*d*) Haller’s layer (large vessels) and (*e*) suprachoroid. The choroidal stroma (*f*) contains various cells including melanocytes (20–30 μm long densely pigmented cells), macrophages, dendritic cells, lymphocytes, non-vascular smooth muscles, intrinsic neurones, nerve fibres associated with vessels and connective tissue elements (i.e. fibroblasts, mast cells, elastic and collagen fibres). **b** DAPI staining showing the nucleus in cells. The nuclei of the retinal pigment epithelial (RPE) cells is clearly seen. The red blood cells within blood vessels and choriocapillaris do not have nucleus (white arrows). **c** Staining for IBA-1 shows a macrophage (red arrow) in a perivascular area within the choroidal stroma. The red blood cells within the blood vessel appear in green due to intrinsic autofluorescence (double white arrow). Scale bars: 50 μm (**a** and **b**), 20 μm (**c**). (Color figure online)

Figure 1 shows the structure of the non-human primate choroid, the heavily pigmented vascular structure of the eye, between the retina and the sclera with its five distinct regions: (a) Bruch’s membrane, (b) choriocapillaris, (c) Sattler’s layer (small, medium sized vessels), (d) Haller’s layer (large vessels) and (e) suprachoroid. It can be seen that the choroidal stroma contains various types of cells including melanocytes with black pigment, melanin. Despite the fact that the animals were bled, there are still some red blood cells in the choroidal blood vessels as seen in Fig. 1b. Immunohistochemical staining for Iba-1 marker identified microglia and macrophages. One macrophage can be seen in the perivascular area in Fig. 1c, with Iba-1 stained in green and the cell nucleus in blue.

### Visualization of iron, zinc, copper and sulphur distribution in young and aged non-human primate choroid by SXRF imaging

**Fig. 2:**
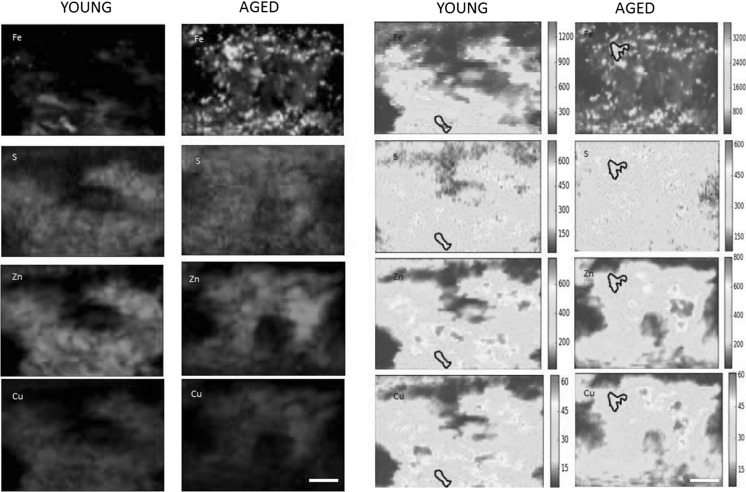
Synchrotron X-ray fluorescence images visualizing iron, sulphur, zinc and copper distributions in a typical example of a young and an aged non-human primate choroid with false colour and heat maps. The colour maps on the two left columns indicate relative concentrations of elements and show the choroid architecture with a diffuse distribution of sulphur, zinc and copper over the majority of the choroidal stroma. Iron, on the other hand, accumulates in small regions. The two right columns show colour gradient heat maps of the same images allowing easier direct qualitative comparison of the trace element concentration. Colour bars correspond to concentration in the units ppm. Note the range of ppm in the iron maps is 0–1400 in the young animal and 0–3400 in the aged animal. Comparing the iron distribution between the young and aged choroid samples, it can be seen that in the aged animal, there are small areas with very high concentration of iron (as “hotspots”) and the map has a granular appearance. It is interesting to note that the areas with high iron content do not correspond with areas of high sulphur, zinc or copper suggesting that accumulation of iron does not coincide with accumulation of these other three elements (scale bar 50 μm)

X-ray fluorescence microscopy revealed the distribution of trace elements in 30 µm thick sections of young and aged non-human primate choroid (Fig. 2). There are similarities between zinc, copper and sulphur maps with diffuse distribution in the choroidal stroma. On the other hand, the iron distribution is different with iron accumulation in hotspots particularly in the aged animals both in the inner and outer choroid. The cellular composition of the choroid is heterogenous, with diverse types of cells, which vary widely in their iron content. Iron accumulations are up to 22 µm in size and seem to be iron-loaded cells with characteristics similar to macrophages.

### Staining with Prussian blue (Perls staining)

**Fig. 3:**
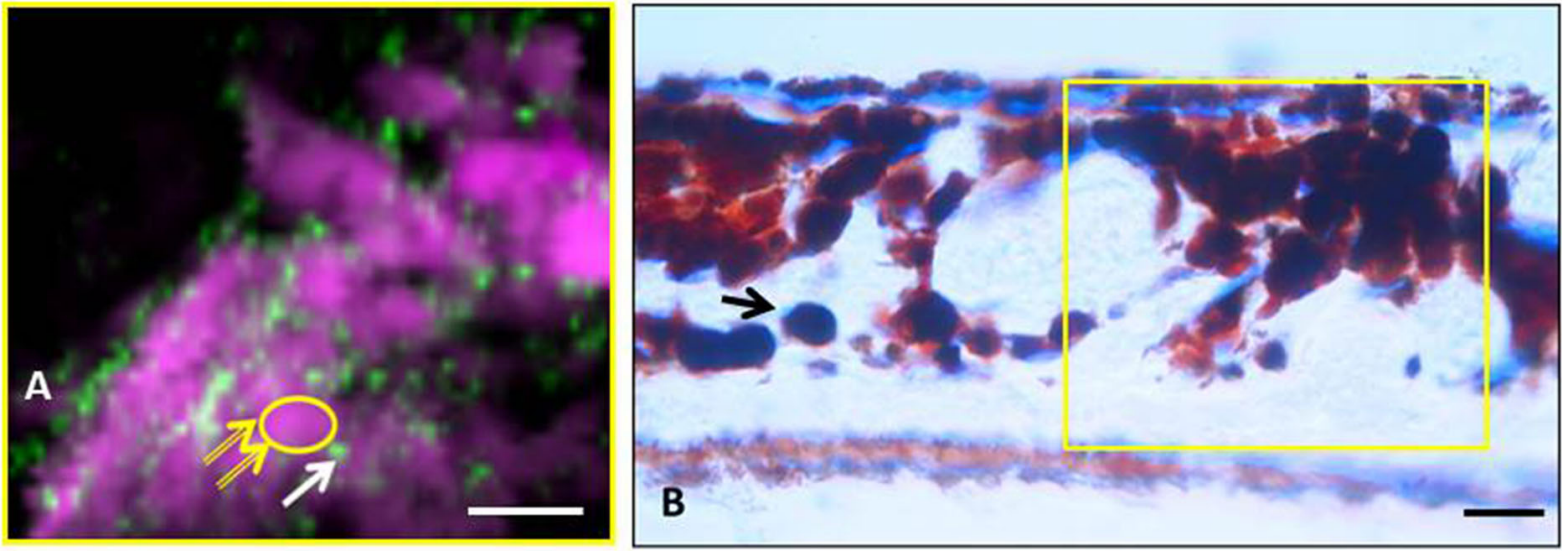
Iron deposition in aged choroidal stroma. **a** A typical aged choroid section showing iron (green) and zinc (pink) X-ray fluorescence (2 μm resolution). **b** Comparison with the adjacent section taken not more than 30 μm from the area shown in A indicates that regions of high zinc intensity correspond to melanocytes. There is correlation between iron fluorescence (in **a**) and Prusian blue (in **b**), which stains Fe^3+^ in ferritin and hemosiderin. PB staining is also seen in apical and basal parts of the retinal pigment epithelium. Similar distribution was observed in bright field image of adjunct section stained with PB. Scale bars: 50 μm (**a**), 20 μm (**b**). (Color figure online)

Comparison of iron fluorescence with optical imaging of Perls staining demonstrated that iron accumulation is preferentially Fe^3+^ (Fig. 3). Perls staining shows non-heme iron deposits in a characteristic blue hue of PB. The method is not quantitative but it is specific to non-heme Fe^3+^.

### Concentration of iron, zinc, copper and sulphur in choroidal stroma and inside blood vessels lumina (from Ugarte et al. 2016)

**Fig. 4:**
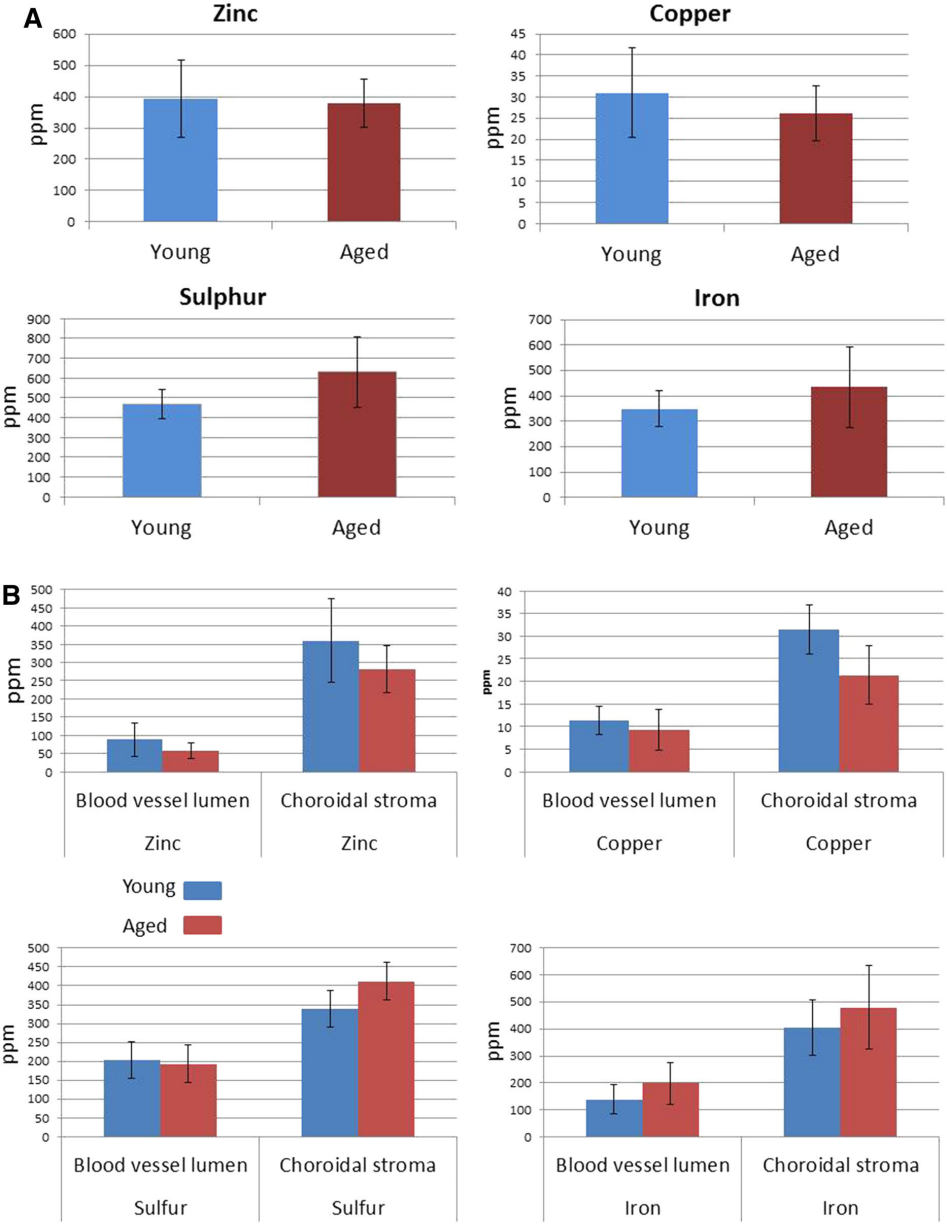
Average metal contents of overall choroidal sections (**a**) and separating blood vessel lumina and choroidal stroma (**b**). Regions of interest (ROI) were manually drawn around the entire choroidal section, inner lumen of blood vessels and choroidal stroma in the X-ray fluorescence false colour maps using ImageJ. The metal contents are expressed as mean ± standard deviation for all animals in each group (n = 4 young, n = 5 aged). We determined that iron and sulphur levels were slightly higher in the choroidal stroma of aged animals, whereas zinc and copper concentrations were lower. None of these differences were statistically significant. Similar changes were found in the contents in the blood vessels lumen suggesting that with age these animals might develop a reduction in copper and zinc levels, while those of iron increase. The concentration of iron in blood is significantly lower than in the choroidal stroma supporting the fact that the iron hotspots are not red blood cells

Iron levels in young and aged choroid were analyzed by measuring averaged iron concentration in the choroidal stroma and within blood vessels (containing red blood cells with heme groups in haemoglobin), as well as iron content in iron-rich accumulations (Fig. 4). There were no significant changes in the overall iron, zinc, copper or sulphur content in the choroidal stroma or in blood vessel lumina comparing young vs aged samples. However, it is striking that the aged choroid is able to accumulate iron selectively in focal regions where the iron fluorescence intensity ranges from 2- to 7-fold (1002–3752 ppm) the mean level in the rest of the choroidal stroma (500 ppm) and maximum iron levels in blood vessel lumina (596 ppm). As shown in Ugarte et al. 2016, iron hotspots with iron ppm > 1000 increased in number (young 4–8 vs. aged 1–93 per area scanned) and size (< 2–13 μm in young animals vs. < 2–22 μm in aged) with age.

### Co-localisation of pairs of trace elements

**Fig. 5:**
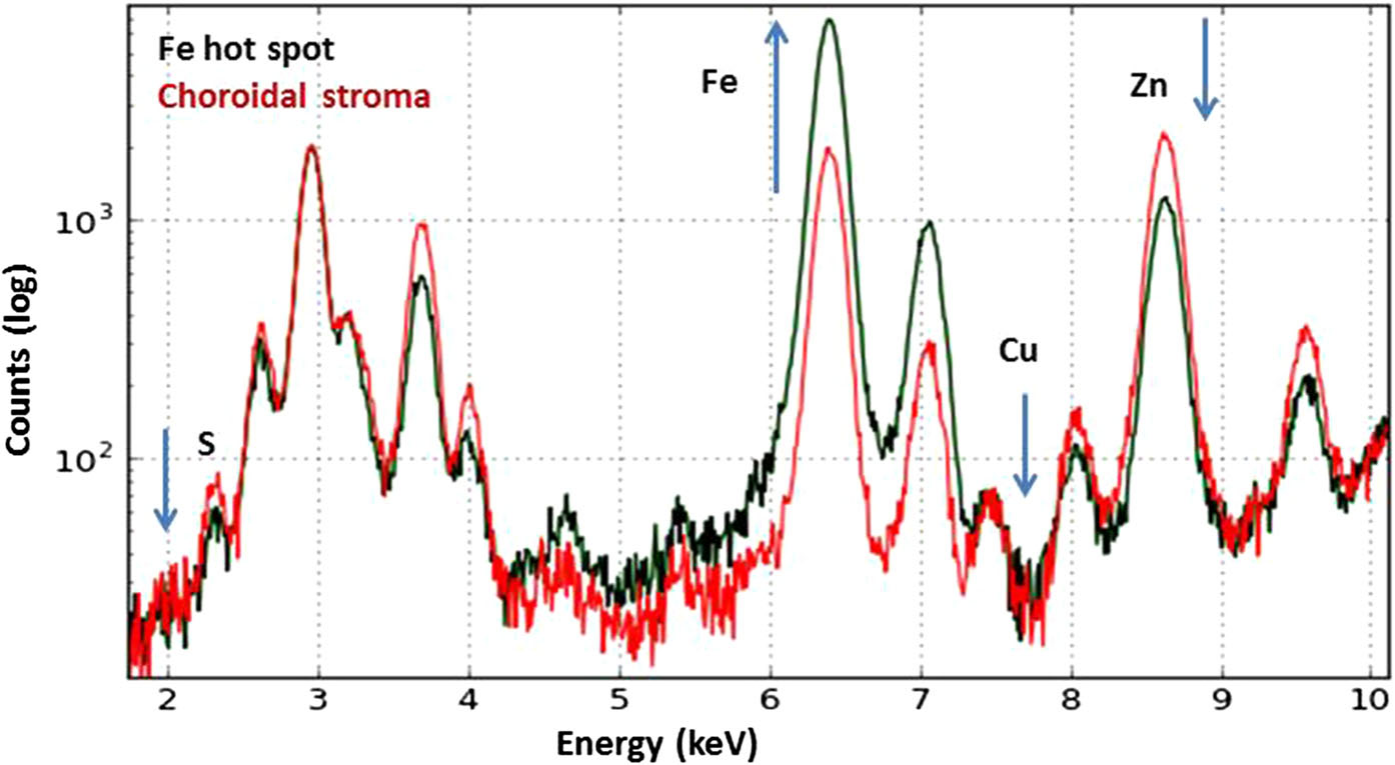
Comparison of the log scale of the X-ray fluorescence spectra obtained from an iron hotspot and a neighbouring area of the choroid, as shown in Fig. 3b (white arrow-hotspot, yellow arrows-choroidal stroma), demonstrates lower concentration of copper, zinc and sulphur in the hotspot compared to the neighbouring choroidal stroma. Spectral deconvolution was done using PyMca. (Color figure online)

Visual comparison of individual element maps shows a clear strong spatial co-localization of copper and zinc within the tissue (Fig. 2). A co-localization is also observed with both copper and sulphur, and zinc and sulphur in the young samples. However, the areas where iron-rich accumulations are found have lower concentrations of zinc and copper than the remaining choroidal stroma (Fig. 5). Co-localisation of the iron signal with sulphur is prominent in young animals but is also lost in areas of increased iron content in aged animals.

### Correlations

**Fig. 6:**
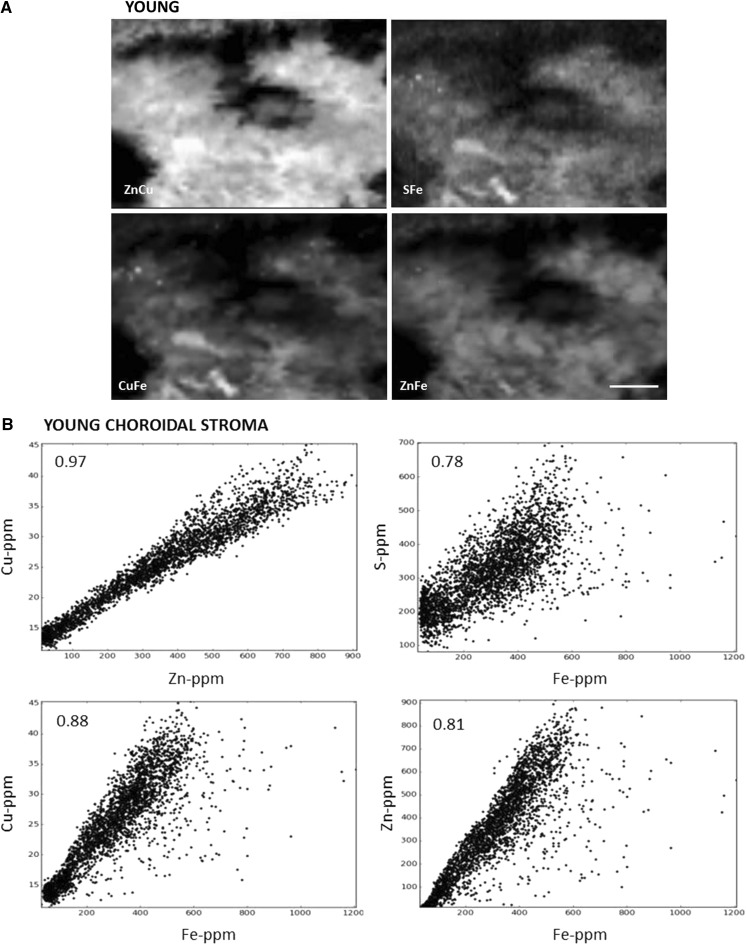

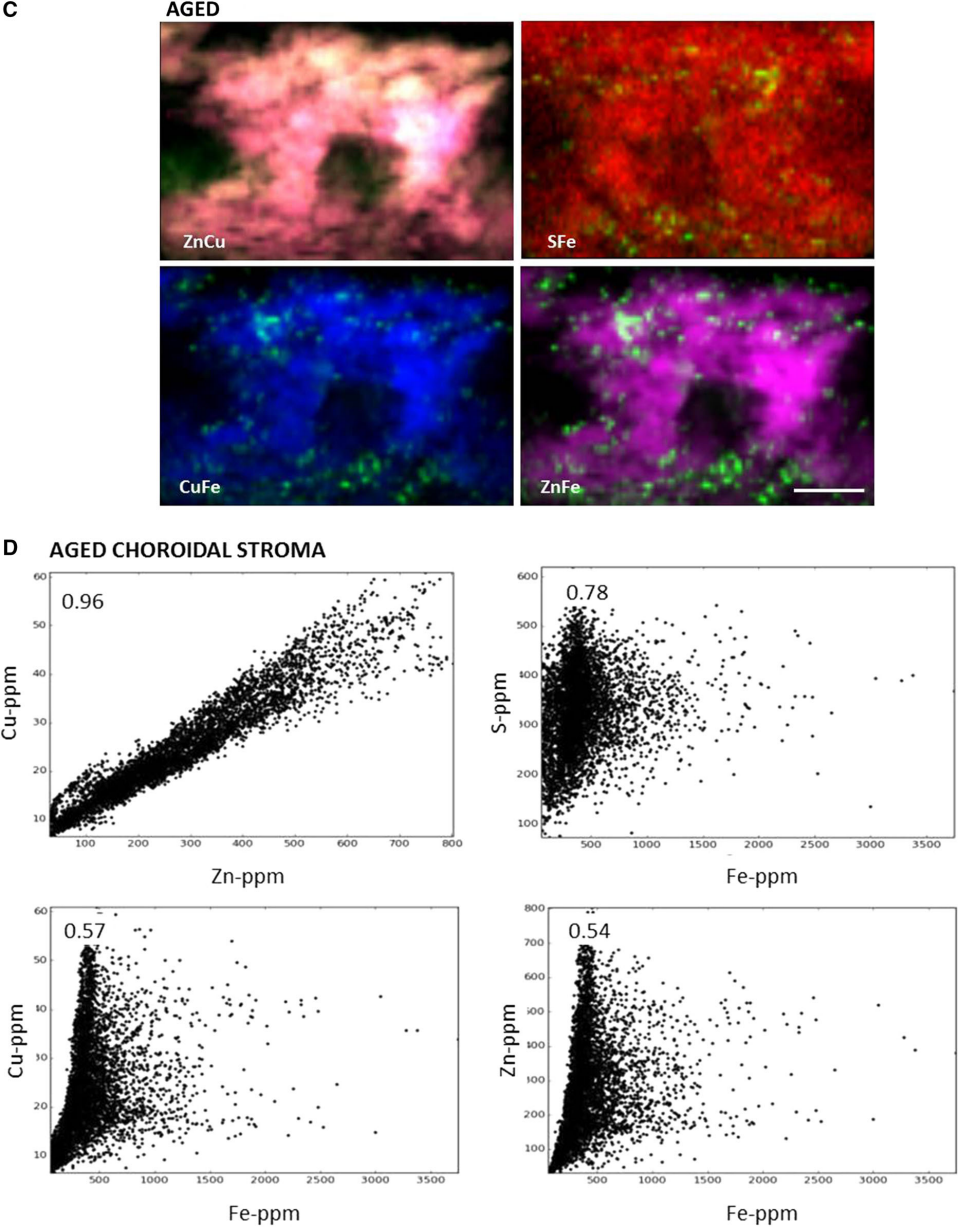

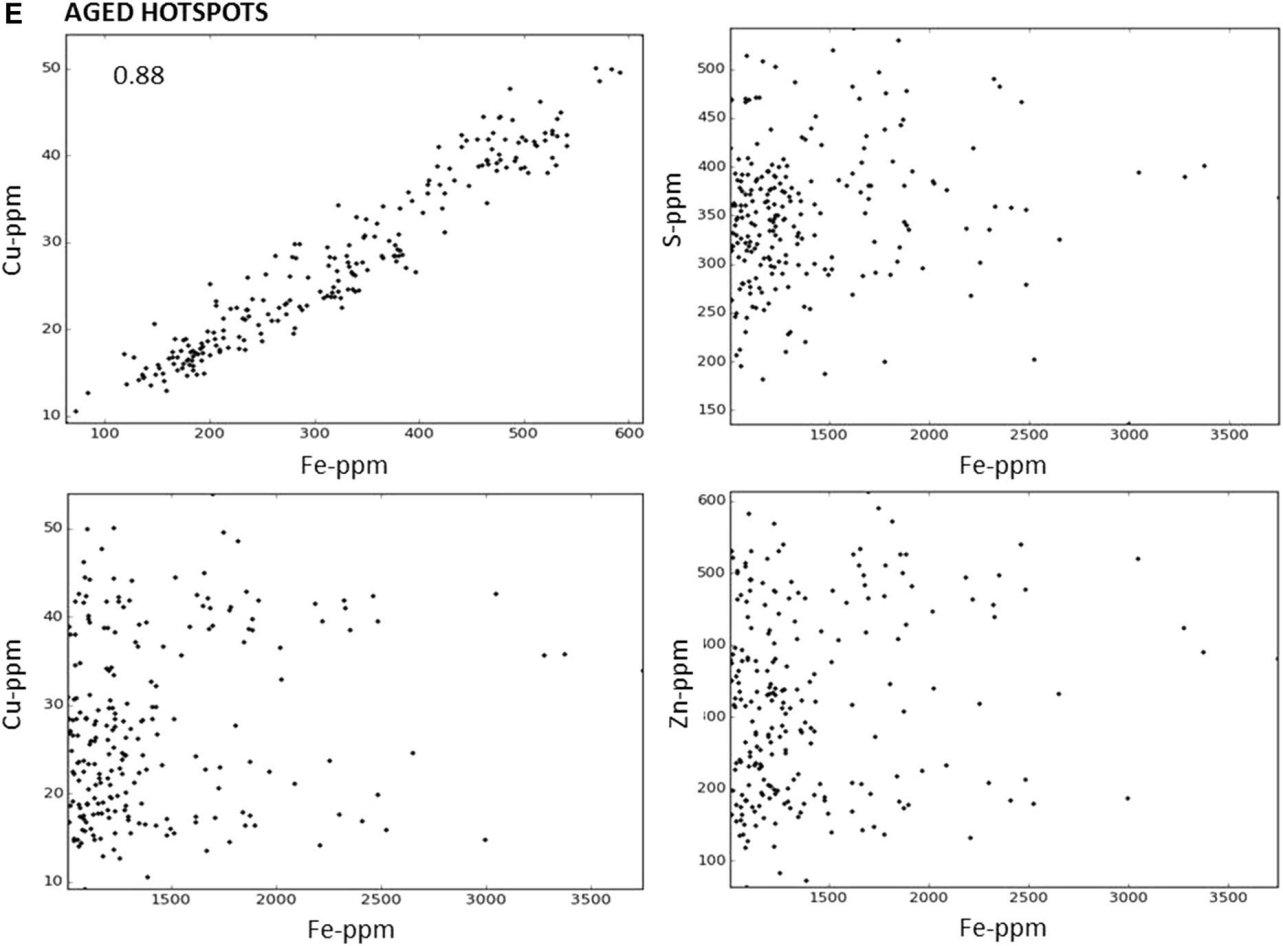
Correlations of pairs of trace elements in the choroid of young and aged male *Macaca fascicularis*. Scatterplots of pixel intensities of young (**a**, **b**) and aged (**c**, **d**, **e**) maps shown in Fig. 2, show that the highest zinc concentrations are correlated with the highest copper concentrations both in young and aged (in the choroidal stroma and iron hotspots). Pearson’s correlation coefficients can be seen in upper left hand corner. This supports the observation that these elements are found together in the choroid of young and aged animals in a similar manner. In young animals there is also a relative co-localisation between iron and zinc, copper and sulphur (**b**). However, there is a change in the ratio of iron to any of the other three trace elements in the aged choroid (**d**) and it is lost in the iron hotspots in aged animals (**e**), with no proportional co-localisation. Scale bars, 50 μm)

Correlations were generated to determine how well the metals co-localised. Sample scatterplots, in which the concentration of two elements are plotted against each other, from SXRF images of one young and one aged animal are illustrated in Fig. 6 with the Pearson correlation coefficients (R) at the top left hand corner. There is a very strong correlation between the localisation of copper and zinc both in young and aged animals. Iron co-localised with zinc and copper to a certain degree in young animals but in the aged samples, the iron hotspots have lower amounts of zinc and copper (Fig. 5). At iron concentration above 1000 ppm (iron hotspots), the correlations with copper, zinc and sulphur are lost (Fig. 6). The lack of proportional codistribution suggests that iron accumulation does not induce a concomitant increase in zinc, copper or zinc-, copper-metalloproteins.

## Discussion

The present study shows focal accumulations of iron in the choroidal stroma of aged old world primate without a concomitant increase in copper, zinc or sulphur. These results extend earlier studies showing increased iron in the retinal pigment epithelium and outer retina of aged humans and experimental animals, with higher increases in women than men (He et al. 2007). In rats, the rises were reported as 3-fold in the RPE (Chen et al. 2009a), 1.3-fold in the neuroretina (Chen et al. 2009b) compared with young animals. It is important to highlight here that the increases found in rat retina by Chen et al. (2009a) did not reflect changes in blood levels, suggesting tissue-specific mechanism.

There are suggestions that the systemic homeostasis of iron, zinc, and copper can change during aging. The average serum ferritin concentrations (Brown 2009; Cals et al. 1994; Cook and Yu 1998; Milman and Schultz-Larsen 1994; Zauber and Zauber 1987), a reflection of body iron stores, and copper blood levels (Belbraouet et al. 2007) have been reported to increase, while plasma and serum zinc to decrease (Haase and Rink 2009). Iron stores in liver and spleen have been found to be elevated in older rats and mice in some studies (Cook and Yu 1998; Massie et al. 1983; Sohal et al. 1999; Takeda et al. 1996), although another has reported no age-related increase (Ahluwalia et al. 2000). In the current study, we did not carry out measurements of trace element systemic metabolism but our observation that the concentration in the iron accumulations is several times higher than the content within blood vessels and surrounding choroidal stroma suggests a facilitated selective uptake and/or retention process rather than an adaptation of the choroid loaded with excess iron.

Previous comparisons carried out by our group between SXRF, ICPMS and Proton induced X-ray emission measurements (Ugarte et al. 2012) demonstrated that the values estimated with PIXE, with concentrations corrected by Rutherford Backscattering Spectrometry, were closer to the ICPMS (“true”) concentrations. The SXRF values were approximately half of the PIXE calculations. The deviation results from the volume reduction of the cryo-sections after deposition on the substrate and the consequent inaccuracy of the nominal thickness section used when modelling the sample in PyMca. This deviation could not be removed completely as the reduction in sample volume does not correspond with the changes in the reference material. Future studies with matrix matched external calibration standards will be considered. The concentrations reported in this study in ppm might not be the “true” concentration in the tissue in situ but the relative increase in the hotspots is reliable.

Further scientific evidence of iron binding/sequestration in choroidal stroma is needed for a better understanding of the biological function and potential toxicity of increased iron levels, as well as the tissue reactions induced by them. We lack information about iron transport, distribution and homeostatic regulation in the choroid. Possible transport pathways into the stroma include absorption from blood vessels or release from melanin in the retinal pigment epithelium and/or choroidal melanocytes. There is no conclusive evidence as to why iron accumulation increased in the aged choroid. It is unclear whether it is a cause or consequence of aging or whether iron homeostasis is perturbed. It could potentially result from: (a) retention in an attempt to achieve detoxification of excess free iron, (b) an adaptive mechanism balancing iron content with the tissue needs or (c) a dysfunction in its homeostatic mechanisms, with increased uptake and/or poor excretion of excess iron. Age-induced retinal iron accumulation in rodents is associated with alterations in homeostatic molecules, with different changes in the RPE/choroid and the neuroretina (Chen et al. 2009a, b; Hahn et al. 2004).

The fact that these accumulations occur in healthy animals suggests it is a normal process of aging. However, continuous accumulation of iron increases the potential for an activated redox state, oxidative stress and tissue damage. Age-related iron accumulation might indeed be a risk factor and a possible contributor for the onset and progression of AMD (Wong et al. 2007).

Comparison of the distribution of the total iron fluorescence in the SXRF maps of aged animals with ferric iron demonstrated by Perls staining showed an anatomical match, suggesting the accumulated iron is mainly Fe^3+^. It is possible that the iron hotspots are ferritin or hemosiderin molecules loaded with Fe^3+^ in stable, insoluble, non-toxic complexes. The lack of concomitant increase in copper in iron hotspots suggests that the choroid in this area does not have a significant oxidative environment. Excess free redox active iron would be expected to be associated with copper sulphur complexes as it has been shown in brain studies (Yoshida et al. 2017). Iron homeostasis is tightly regulated by cuproproteins and an enhanced turnover has been demonstrated to be adaptively induced against excess free reactive iron (Yoshida et al. 2017). Low sulphur/iron correlation in the iron hotspots suggest iron is bound in the form of multimetal clusters such as ferritin. A fully loaded molecule of ferritin can contain up to 4500 atoms of iron (mostly Fe^3+^). The storage of iron within ferritin may act as a protective mechanism, but heavily loaded ferritin may eventually produce free radicals and oxidative stress.

The iron hotspots were found in regions where there are, in fact, lower signals of copper and zinc. This raises the possibility that iron accumulation might be the consequence of focal zinc or copper deficiency in specific cells. The cellular homeostases of iron, zinc, and copper are closely interlinked. Copper is necessary for the activity of ceruloplasmin and hephaestin and facilitates iron excretion by ferroportin. Ceruloplasmin and hephaestin, acting as ferroxidases, convert Fe^2+^–Fe^3+^, generate an iron gradient, and facilitate export of iron and uptake by proteins. The absence of ceruloplasmin can result in retinal iron accumulation (Bonaccorsi di Patti et al. 2018; Zheng et al. 2018). Zinc-deficient animals accumulate iron in several organs (Himoto and Masaki, 2018). Numerous genes respond to alterations in cellular zinc levels with changes in mRNA levels. An in vitro study has shown that zinc deficiency results in increased levels of iron regulatory proteins and could raise cellular iron uptake and hence intracellular levels.

It is important to emphasize here that the thickness of our sections was 30 µm at the time of cutting (although considerably thinner after drying) and the 2-D projected image is generated by all the tissue in the path of the beam in the section volume. The maximum intensity value for each x,y position being the collection of all the structures within the sample volume at that position. In some areas, there might be partial overlap of some cells. Our method, therefore, does not allow us to confirm whether two trace elements detected in the same projected pixel are located in the same physical structure or on two distinct structures in a 3-D volume. The correlations are nevertheless a useful tool to gain an idea of underlying interactions and tissue reactions. We decided to use this section thickness after preliminary tests. Thinner sections with reduced sample volume resulted in very low fluorescence signal (low signal-to-noise) despite long dwell times, which were inaccurate and not suitable for our study.

Further studies are necessary to establish whether iron-accumulation in the aged choroid is a cause or an effect of changes in the homeostases of other trace elements and/or other age-related processes (e.g. inflammation) and the potential contribution to the pathogenesis of AMD.
